# PReS-FINAL-2115: Differences in musculoskeletal clinical expression between Spanish and Mexican patients with juvenile spondyloarthritis: data from the Mexespa project

**DOI:** 10.1186/1546-0096-11-S2-P127

**Published:** 2013-12-05

**Authors:** WA Sifuentes Giraldo, CA Guillén Astete, ML Gámir Gámir, C Arnal Guimerá, D Clemente Garulo, I Calvo, I Rotes, J Sampedro Álvarez, J García Consuegra, M Medrano, P Collado, R Roldán, S Bustabad

**Affiliations:** 1 Rheumatology, University Hospital Ramon y Cajal, Madrid, Spain; 2 Rheumatology, Hospital Vall d'hebron, Barcelona, Spain; 3 Hospital del Niño Jesus, Madrid, Spain; 4 Hospital la Fé, Valencia, Spain; 5 Hospital san Rafael, Barcelona, Spain; 6 Rheumatology, Hospital Virgen de la Salud, Toledo, Spain; 7 Rheumatology, Hospital la Paz, Madrid, Spain; 8 Rheumatology, Hospital Miguel Servet, Zaragoza, Spain; 9 Rheumatology, Hospital Severo Ochoa, Madrid, Spain; 10 Rheumatology, Hospital Reina Sofia, Córdoba, Spain; 11 Rheumatology, Hospital de la Laguna, Tenerife, Spain

## Introduction

Juvenile spondyloarthritis (J-spa) is characterized by a predominantly peripheral involvement and more frequent presentation as undifferentiated forms. However, like its adult-onset equivalent, it has the potential to develop structural damage and evolve to juvenile ankylosing spondylitis (J-AS), with consequent functional impairment that will last for the lifetime of the patient. Although descriptions of jspa in worldwide different populations are scarce, it has been suggested that clinical expression could be different in Spanish and Mexican patients.

## Objectives

Compare the patterns of musculoskeletal involvement in J-spa patients from Spain and Mexico.

## Methods

We conducted a descriptive study based on data of mexespa study, which consists of binational cohort distributed in 10 Spanish centers and 6 Mexican. Analyzed data were demographics, musculoskeletal manifestations, metrology and diagnosis.

## Results

A total of 128 patients were included, 95 from Spain and 33 from Mexico. 65% of Spanish patients were boys and 85% in Mexico. Mean age of onset was higher in Mexican group (12 ± 3 years) than Spanish group (10 ± 4 years), with a longer disease duration ofthe latter group. 94% of Spanish patients were Caucasian, while in Mexico predominated white-Indian (64%). Frequency of HLA-B27 was higher in Spain (47%) than Mexico (27%), as well as family history of spa (45% and 18%, respectively). Almost all patients in both countries had some type of musculoskeletal symptom at the onset (95% in Spain and 94% in Mexico). However, the patterns were different between both countries. In Spain predominated peripheral arthritis affecting lower extremities and peripheral enthesitis in Mexico. In Spain 44% had peripheral entheses affected and 9% axial entheses, but these percentages were higher in Mexico (36% and 85%, respectively). Mean modified Schober was 6 cm in both groups and no other significant differences were found in metrology. There were also no significant differences in C-HAQ, BASDAI and BASFI indexes, although these values were slightly higher in Mexico. More patients in the Mexican cohort were diagnosed with J-AS in Mexico (61%) than in the Spanish (7%), but undifferentiated J-spa and psoriatic arthritis were more frequent in the Spanish patients (46% and 36%, respectively) than in Mexican patients (36% and 3%, respectively).

## Conclusion

Results show a different patterns of musculoskeletal involvement in Spanish and Mexican J-spa. Differences could be related to several aspects including social and environmental factors, age of onset, genetics and different J-spa subtypes, and the follow-up of those patients during 5 years will permit define better what factors are associated with worse outcome in Mexican patients.

## Disclosure of interest

None declared.

